# Near-infrared venous imaging may be more useful than ultrasound guidance for novices to obtain difficult peripheral venous access: A crossover simulation study

**DOI:** 10.1097/MD.0000000000033320

**Published:** 2023-03-24

**Authors:** Shinichiro Sekiguchi, Kiyoshi Moriyama, Joho Tokumine, Alan Kawarai Lefor, Harumasa Nakazawa, Yasuhiko Tomita, Tomoko Yorozu

**Affiliations:** a Department of Medical Education, Kyorin University School of Medicine, Mitaka, Tokyo, Japan; b Department of Anesthesiology, Kyorin University School of Medicine, Mitaka, Tokyo, Japan; c Department of Surgery, Jichi Medical University, Shimotsuke, Tochigi, Japan.

**Keywords:** near-infrared vascular imaging, simulation training, ultrasound guidance, venous access

## Abstract

Difficult peripheral venous access, especially in obese people, is challenging for novices. We conducted a randomized cross-over study to examine whether near-infrared venous imaging or ultrasound guidance is more useful for novice operators to obtain difficult peripheral venous access.

**Methods:**

Medical students were recruited as participants. After receiving basic training using commercial simulators, participants were randomly assigned to obtain simulated venous access using a difficult venous access simulator with near-infrared venous imaging or ultrasound guidance in a randomized cross-over design. A difficult venous access simulator was newly developed with deep and narrow vessels to simulate an obese patient. The primary outcome measure of the study was the first-time success rate (%), and the secondary outcome measures included procedure time (seconds) and the number of 3 consecutive successful attempts, to represent proficiency with the procedure. Pearson chi-square test, the Wilcoxon signed-rank test, and generalized estimating equations were used for statistical analysis.

**Results:**

Forty-one medical students with no experience performing peripheral venous access were enrolled in this study. The rate of successful first attempts did not differ between the 2 groups (70% for near-infrared; 65% for ultrasound guidance; *P* = .64). The duration of the procedure for the first attempt was significantly shorter using near-infrared imaging (median: 14; interquartile range: 12–19) compared to ultrasound guidance (median 46; interquartile range: 26–52; *P* = .007). The number of attempts until 3 consecutive successes was not significantly different comparing the 2 approaches (near-infrared: 3 (3, 7.25), ultrasound guidance: 3 (3, 6.25), *P* = .63).

**Conclusion:**

There was no difference in success rate of first-time attempts or acquiring proficiency for the 2 methods. However, duration of the first attempt was significantly shorter with near-infrared imaging than with ultrasound guidance. Near-infrared imaging may require less training than ultrasound guidance. Near-infrared venous imaging may be useful for novices to obtain difficult peripheral venous access in obese patients.

## 1. Introduction

Peripheral venous catheters are the most commonly used devices for vascular access in routine clinical practice. Obesity is associated with difficult peripheral venous access.^[1]^ The number of obese people is increasing rapidly worldwide.^[2]^ If this trend continues, difficulties obtaining peripheral venous access in clinical practice will also increase. Recently, the usefulness of ultrasound guidance to obtain peripheral venous access has been reported.^[3]^ However, special training is required to master the technique for ultrasound-guided vascular access.^[4,5]^ Another report states that imaging peripheral blood vessels with near-infrared light facilitates peripheral venous access.^[6]^ This study examined whether ultrasound guidance or near-infrared light is more useful for novice operators to obtain difficult peripheral venous access due to obesity.

## 2. Materials and methods

The present study was approved by the Faculty of Medicine Research Ethics Committee, Kyorin University (approval number 1804) and registered in the University Hospital Medical Information Network Center Clinical Registration System (UMIN000045269). The study was conducted in accordance with Consolidated Standards of Reporting Trials guidelines.

The study was designed as a randomized, prospective crossover study. Participants were recruited from among fourth and fifth-year medical students as volunteers. Exclusion criteria included experience of obtaining peripheral venous access in a patient, and refusal to participate. Written informed consent was obtained from all participants. Participant recruitment and data collection were performed from September 2021 to November 2022.

### 2.1. Simulation training

Before starting the study, simulation training was conducted. The Intravenous ARM III (Kyoto Kagaku Co., Japan) simulator was used to train the direct visual and palpation techniques for peripheral venous access for 30 minutes. The peripheral venous catheter was BD Insight 22G, 25 mm (Nippon Becton Dickinson Co., Japan). A brief explanation of the principles of near-infrared venous imaging was given, and each participant searched for a vein in his or her own arm using near-infrared vein imaging. The near-infrared visualization system was Vein Viewer Flex (Terumo Co., Japan).

Next, the principles of ultrasound guidance were briefly explained, followed by a demonstration of ultrasound imaging of veins in the upper extremities. Ultrasound imaging was performed with a Sonotore Linear (linear probe 7.5 MHz, ALFABIO Co., Japan). Out-of-plane (dynamic needle tip positioning^[7]^) and in-plane approaches were introduced as ultrasound vascular access techniques.^[4,5,8]^ A 1-hour practical training session was held using the UGP GEL (AGL800, Alfabio Co., Japan).^[9–11]^ This simulator uses a simulated blood vessel 5 mm in diameter and 5 mm deep, embedded in an ultrasound-transparent gel. After simulation training, participants were randomly assigned, using a sealed envelope system, to perform simulated vascular access using near-infrared vein imaging or ultrasound guidance using a “difficult venous access simulator.”

### 2.2. Difficult venous access simulator

**Figure 1.**
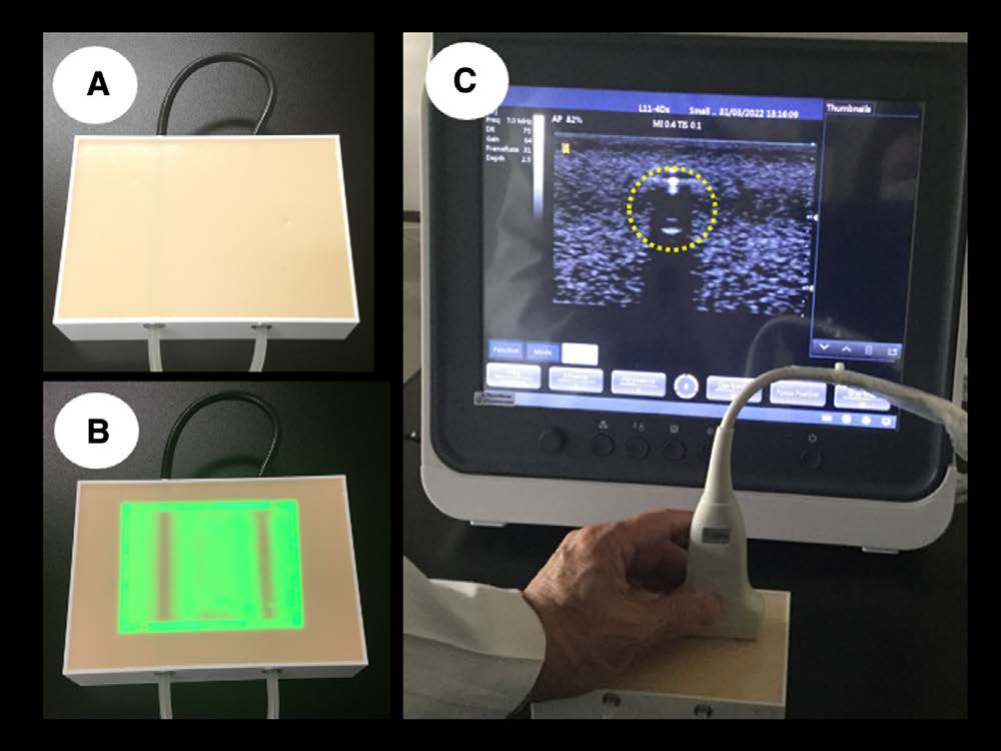
Difficult venous access simulator. (a) Simulated vessels cannot be seen in the simulator with the eye. (b) Near-infrared imaging with the simulator, allowing 1 to see simulated vessels. (c) Ultrasound imaging with the simulator providing a view of simulated vessels.

A simulator was developed especially for this study (Kyoto Kagaku Co., Japan) (Fig. 1). The new simulator used a mixture of urethane gel and epoxy resin (mainly fatty acid esters) to simulate the soft tissues of a limb. In addition, urethane paint was applied to the surface to reduce stickiness. Simulated blood vessels were made with condensed silicone rubber and painted with black pigment on the surface. In this simulator, blood vessels with an inner diameter of 3 mm and a depth of 5 mm are simulated by embedding them in a material that transmits near-infrared light and ultrasound waves. The blood vessels cannot be seen with the naked eye in this simulator. The inner diameter of the simulated blood vessel in this simulator is smaller (3 mm in diameter) than in the simulator used for training, making the puncture more difficult.

Punctures continued until 3 consecutive successful attempts were made. Even if 3 consecutive successful attempts were not achieved, the trial was terminated after 20 attempts. Three consecutive attempts were hypothesized to represent proficiency with the technique. Success was defined when water in the lumen of the simulated vessel was aspirated from the catheter after placement. Failure was defined as failure to place the catheter into the simulated vessel within 3 minutes, or not to achieve 3 consecutive successes within 20 attempts. The procedure time was defined as the time from the start of puncture to confirmation of water backflow from the simulated vessel. Participants performed the puncture using the assigned method (near-infrared or ultrasound), then performed using the other method (crossover study).

The primary outcome measure of the study was the rate of successful first attempts, and the secondary outcome measures included procedure time and the number of 3 consecutive successful attempts. Data were electronically stored in an anonymized manner.

### 2.3. Statistical analysis

Success rates were expressed as percentages (%). Procedure times (seconds) and number of 3 consecutive successes were shown as median (first quartile, third quartile). The presence or absence of carryover effects was evaluated with the Mann–Whitney *U* test. Pearson chi-square test was used to compare success rates. The Wilcoxon signed-rank test was used to compare procedure times. Three consecutive successes were analyzed with the generalized estimating equations, using a binomial distribution, and logit for link function. Data was analyzed with EZR statistical software (Saitama Medical Center, Jichi Medical University, Saitama, Japan).^[12]^ A *P* value < .05 was considered statistically significant.

### 2.4. Power analysis

At the time the present study began, there were no comparative studies comparing near-infrared vein imaging and ultrasound guidance in adult patients. The clinical difference between the 2 techniques could not be determined. Therefore, assuming a clinically significant difference, the sample size required for 80% power at *ɑ* = 0.05 was estimated to be 30 participants. In this study, 40 medical students were enrolled to account for possible drop-outs or exclusions.

## 3. Results

**Figure 2.**
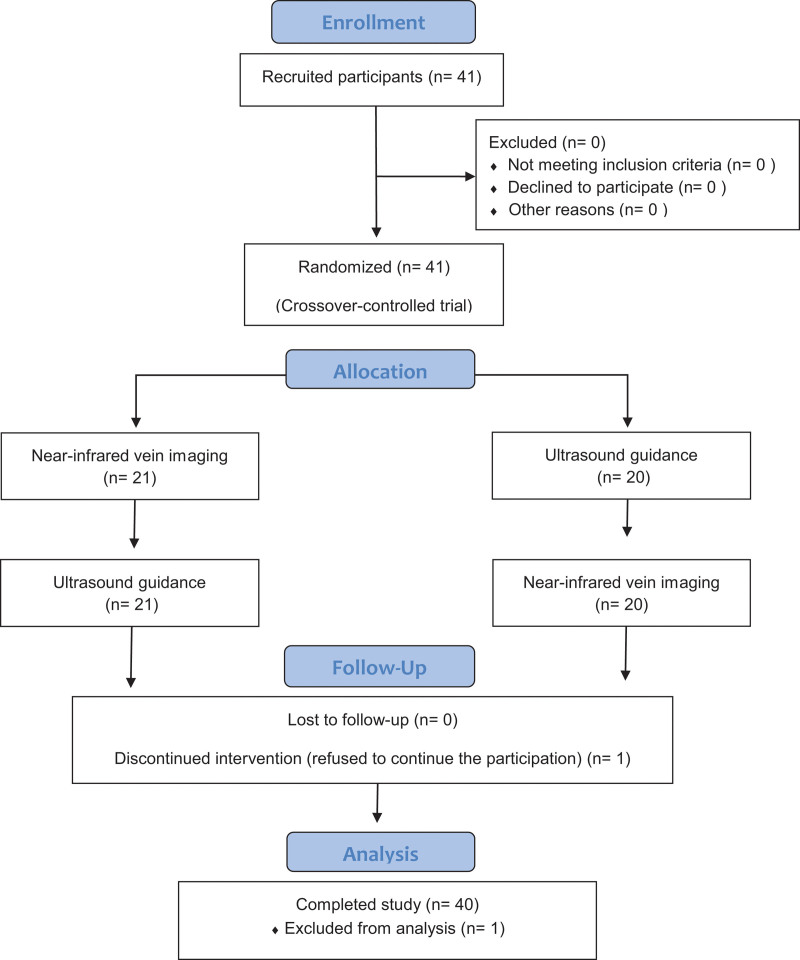
CONSORT diagram. CONSORT = consolidated standards of reporting trials.

Forty-one medical students participated in the present study. One participant data was excluded due to missing data (refusal to continue participation due to fatigue, Fig. 2). The carryover effect was not statistically significant (success rates: 0.70, procedure time for the first attempt: 0.59, 3 consecutive successful attempts: 0.72). The first attempt success rate comparing near-infrared venous imaging and ultrasound guidance showed no significant difference (near-infrared: 70%, ultrasound 65%, *P* = .64). Procedure time for the first attempt was significantly shorter with near-infrared vein imaging compared to ultrasound guidance (near-infrared vein: 14 (12, 19), ultrasound: 45 (26, 52), *P* = .007). The numbers of 3 consecutive successful attempts were not significantly different between the 2 methods (near-infrared vein imaging: 3 (3, 7.25), ultrasound guidance: 3 (3, 6.25), generalized estimating equations: comparing odds ratios, *P* = .63).

## 4. Discussion and conclusions

In the present study, we investigated whether near-infrared venous imaging or ultrasound guidance facilitates novices in obtaining difficult peripheral venous access using a simulated vein. The results show that obtaining difficult vascular access with near-infrared venous imaging was faster than when using ultrasound guidance, with no difference in success rate or proficiency using the technique. The simulator used in this study (a difficult venous access simulator) was designed to mimic the conditions under which veins are invisible to the naked eye, similar to the situation in some obese patients. The authors believe that near-infrared venous imaging may be useful for securing peripheral venous access in obese patients. Near-infrared venous imaging also has the advantage of requiring less training than ultrasound guidance.

Near-infrared venous imaging was expected to improve the success rate for obtaining peripheral venous access in children.^[13–21]^ Most studies show that the effectiveness of near-infrared venous imaging is limited. Curtis et al compared near-infrared venous imaging and ultrasound guidance in children and found no advantage for either method over conventional device placement.^[22]^ Aulagnier et al studied the usefulness of near-infrared venous imaging in adults and found no benefit.^[23]^ Kumar et al also studied adult patients and showed that near-infrared venous imaging increased only the success rate for the initial attempt.^[24]^

Recently, Yalçinli et al compared near-infrared venous imaging, ultrasound guidance, and conventional methods in adult patients and found that ultrasound guidance had a higher initial attempt success rate than near-infrared venous imaging or conventional methods.^[25]^ In that study, participants were knowledgeable and experienced in ultrasound guidance and near-infrared vein imaging before the study. Furthermore, participants were assessed for mastery of skills prior to data collection, and if the skill did not reach a predefined “mastery level,” an additional 4 hours of teaching, including simulation training, was added. The study by Yalçinli et al showed that with adequate clinical and simulation training, ultrasound guidance may have a higher success rate than other methods. However, the present study shows that near-infrared venous imaging is more useful than ultrasound guidance for novices. A limitation of the present study is the difference in fidelity between the simulator and a patient. The influence of simulator fidelity requires further study.

Cannulating veins using ultrasound guidance requires appropriate training and improves with experience. However, no consensus of a clear goal for technical skills acquisition has been established. In the present study, the simulation training given to participating medical students had the same content as preclinical basic training for residents.^[9–11]^ The education is a competency-based modular system designed to allow participants to acquire the basic skills for vascular access, which is constructed in a step-by-step manner.^[9]^ Certain aspects of the effectiveness of this education system have been proven.^[10,11]^ However, this system targets the acquisition of basic skills and does not guarantee success in more difficult vascular access. It was hypothesized that ultrasound guidance after simulation training using this education system would have a higher success rate and improved skills acquisition compared to near-infrared venous imaging, but the results show that this hypothesis was rejected. The development of skill-acquisition programs for ultrasound-guided peripheral venous cannulation in difficult cases may require advanced skills and the need to set higher goals for skill acquisition.

Ultrasound guidance has a great advantage over near-infrared venous imaging in that it can identify deeper veins. Near-infrared venous imaging has a limitation in its ability to locate deep veins, which cannot be observed over 5 mm deep. For this reason, ultrasound guidance is more clinically useful than near-infrared venous imaging. However, appropriate education is critical for this purpose. Near-infrared venous imaging may be useful as a method that requires less education and a useful way for novices to gain experience.

Many studies have evaluated the teaching methods used for ultrasound guided venous access, but there is still no method accepted as the optimal approach.^[26]^ In conclusion, near-infrared venous imaging may be a useful alternative to ultrasound guidance to obtain difficult peripheral venous access in obese patients, especially for novices.

## Acknowledgments

The authors thank Ms. Okada (Laboratory assistant, Division of Biological Function Research) for her assistance.

## Author contributions

**Conceptualization:** Shinichiro Sekiguchi, Joho Tokumine.

**Data curation:** Shinichiro Sekiguchi, Joho Tokumine.

**Formal analysis:** Shinichiro Sekiguchi, Kiyoshi Moriyama, Harumasa Nakazawa.

**Investigation:** Shinichiro Sekiguchi, Joho Tokumine.

**Methodology:** Shinichiro Sekiguchi, Kiyoshi Moriyama.

**Project administration:** Kiyoshi Moriyama, Joho Tokumine, Yasuhiko Tomita.

**Supervision:** Tomoko Yorozu.

**Validation:** Tomoko Yorozu.

**Writing – original draft:** Shinichiro Sekiguchi, Kiyoshi Moriyama, Joho Tokumine.

**Writing – review and editing:** Alan Kawarai Lefor, Kiyoshi Moriyama.

## References

[1] Sebbane M Claret PG Lefebvre S. Predicting peripheral venous access difficulty in the emergency department using body mass index and a clinical evaluation of venous accessibility. J Emerg Med. 2013;44:299–305. 22981661 10.1016/j.jemermed.2012.07.051

[2] Abarca-Gómez L Abdeen ZA Hamid ZA. Worldwide trends in body-mass index, underweight, overweight, and obesity from 1975 to 2016: a pooled analysis of 2416 population-based measurement studies in 128·9 million children, adolescents, and adults. Lancet. 2017;390:2627–42. 29029897 10.1016/S0140-6736(17)32129-3 PMC5735219

[3] van Loon FHJ Buise MP Claassen JJF. Comparison of ultrasound guidance with palpation and direct visualisation for peripheral vein cannulation in adult patients: a systematic review and meta-analysis. Br J Anaesth. 2018;121:358–66. 30032874 10.1016/j.bja.2018.04.047

[4] Lamperti M Biasucci DG Disma N. European Society of Anaesthesiology guidelines on peri-operative use of ultrasound-guided for vascular access (PERSEUS vascular access). Eur J Anaesthesiol. 2020;37:344–76. 32265391 10.1097/EJA.0000000000001180

[5] Moureau N Lamperti M Kelly LJ. Evidence-based consensus on the insertion of central venous access devices: definition of minimal requirements for training. Br J Anaesth. 2013;110:347–56. 23361124 10.1093/bja/aes499

[6] Zhang Z Wang X Zhang L. Infrared vein imaging for insertion of peripheral intravenous catheter for patients requiring isolation for severe acute respiratory syndrome coronavirus 2 infection: a nonrandomized clinical trial. J Emerg Nurs. 2022;48:159–66. 35115182 10.1016/j.jen.2021.10.001 PMC8506227

[7] Bai B Tian Y Zhang Y. Dynamic needle tip positioning versus the angle-distance technique for ultrasound-guided radial artery cannulation in adults: a randomized controlled trial. BMC Anesthesiol. 2020;20:231. 32928119 10.1186/s12871-020-01152-1 PMC7491138

[8] Safety Committee of Japanese Society of Anesthesiologists. Practical guide for safe central venous catheterization and management 2017. J Anesth. 2020;34:167–86. 31786676 10.1007/s00540-019-02702-9 PMC7223734

[9] Asao T. CVC Instructor’s Guide ver. 4. Japanese Association for Medical Simulation; 2018. Available at: http://jams.kenkyuukai.jp/images/sys/information/20181213093339-935A51034B32F5111FD8DE655ED0B884BC1B33A7F92A346AC05BE6078DF323DB.pdf [access date February 22, 2023].

[10] Kikuchi M Asao T Tokumine J. A novel system for teaching the in-plane vascular access technique: a simulation study. Medicine (Baltim). 2021;100:e27201. 10.1097/MD.0000000000027201 PMC8448066 34664850

[11] Sugiki D Matsushima H Asao T. A web-based self-learning system for ultrasound-guided vascular access. Medicine (Baltim). 2022;101:e31292. 10.1097/MD.0000000000031292 PMC9622633 36316890

[12] Kanda Y. Investigation of the freely available easy-to-use software “EZR” for medical statistics. Bone Marrow Transplant. 2013;48:452–8. 23208313 10.1038/bmt.2012.244 PMC3590441

[13] Simhi E Kachko L Bruckheimer E. A vein entry indicator device for facilitating peripheral intravenous cannulation in children: a prospective, randomized, controlled trial. Anesth Analg. 2008;107:1531–5. 18931210 10.1213/ane.0b013e318185cdab

[14] Chapman LL Sullivan B Pacheco AL. VeinViewer-assisted Intravenous catheter placement in a pediatric emergency department. Acad Emerg Med. 2011;18:966–71. 21854488 10.1111/j.1553-2712.2011.01155.x

[15] Kim MJ Park JM Rhee N. Efficacy of VeinViewer in pediatric peripheral intravenous access: a randomized controlled trial. Eur J Pediatr. 2012;171:1121–5. 22415409 10.1007/s00431-012-1713-9

[16] Kaddoum RN Anghelescu DL Parish ME. A randomized controlled trial comparing the AccuVein AV300 device to standard insertion technique for intravenous cannulation of anesthetized children. Paediatr Anaesth. 2012;22:884–9. 22694242 10.1111/j.1460-9592.2012.03896.x

[17] de Graaff JC Cuper NJ Mungra RA. Near-infrared light to aid peripheral intravenous cannulation in children: a cluster randomised clinical trial of three devices. Anaesthesia. 2013;68:835–45. 23763614 10.1111/anae.12294

[18] Cuper NJ de Graaff JC Verdaasdonk RM. Near-infrared imaging in intravenous cannulation in children: a cluster randomized clinical trial. Pediatrics. 2013;131:e191–7. 23230072 10.1542/peds.2012-0968

[19] Rothbart A Yu P Müller-Lobeck L. Peripheral intravenous cannulation with support of infrared laser vein viewing system in a pre-operation setting in pediatric patients. BMC Res Notes. 2015;8:463. 26391665 10.1186/s13104-015-1431-2 PMC4576370

[20] Park JM Kim MJ Yim HW. Utility of near-infrared light devices for pediatric peripheral intravenous cannulation: a systematic review and meta-analysis. Eur J Pediatr. 2016;175:1975–88. 27785562 10.1007/s00431-016-2796-5

[21] Saju AS Prasad L Reghuraman M. Use of vein-viewing device to assist intravenous cannulation decreases the time and number of attempts for successful cannulation in pediatric patients. Paediatr Neonatal Pain. 2019;1:39–44. 35548377 10.1002/pne2.12009 PMC8975231

[22] Curtis SJ Craig WR Logue E. Ultrasound or near-infrared vascular imaging to guide peripheral intravenous catheterization in children: a pragmatic randomized controlled trial. CMAJ. 2015;187:563–70. 25897047 10.1503/cmaj.141012 PMC4435867

[23] Aulagnier J Hoc C Mathieu E. Efficacy of AccuVein to facilitate peripheral intravenous placement in adults presenting to an emergency department: a randomized clinical trial. Acad Emerg Med. 2014;21:858–63. 25176152 10.1111/acem.12437

[24] Kumar A Negi M Khanka J. Initial experience with use of infrared assistance for intravenous injection of radiopharmaceuticals. World J Nucl Med. 2020;20:172–5. 34321970 10.4103/wjnm.WJNM_86_20 PMC8286008

[25] Yalçinli S Karbek Akarca F Can O. Comparison of standard technique, ultrasonography, and near-infrared light in difficult peripheral vascular access: a randomized controlled trial. Prehosp Disaster Med. 2022;37:65–70. 34865664 10.1017/S1049023X21001217

[26] Okano H Mayumi T Kataoka Y. Outcomes of simulation-based education for vascular access: a systematic review and meta-analysis. Cureus. 2021;13:e17188. 34414052 10.7759/cureus.17188 PMC8365863

